# Clinical characteristics of optic neuritis phenotypes in a 3-year follow-up Chinese cohort

**DOI:** 10.1038/s41598-021-93976-1

**Published:** 2021-07-16

**Authors:** Chaoyi Feng, Qian Chen, Guixian Zhao, Zhenxin Li, Weimin Chen, Yan Sha, Xinghuai Sun, Min Wang, Guohong Tian

**Affiliations:** 1 Department of Ophthalmology, Eye Ear Nose and Throat Hospital of Fudan University, 83 Fenyang Road, Shanghai, 200031 China; 2 Department of Neurology, Huashan Hospital, Fudan University, Shanghai, China; 3 Department of Neurology, Shanghai Deji Hospital, Shanghai, China; 4 Department of Radiology, Eye Ear Nose and Throat Hospital of Fudan University, Shanghai, China; 5 State Key Laboratory of Medical Neurobiology, Institutes of Brain Science, Fudan University, Shanghai, China

**Keywords:** Neurology, Eye diseases, Neurological disorders

## Abstract

To evaluate the clinical characteristics of optic neuritis (ON) with different phenotypes. This prospective study recruited patients with new-onset ON between January 2015 and March 2017 who were followed-up for 3 years. They were divided into the myelin oligodendrocyte glycoprotein-seropositive (MOG-ON), aquaporin-4-seropositive (AQP4-ON), and double-seronegative (seronegative-ON) groups, and their clinical characteristics and imaging findings were evaluated and compared. Two-hundred-eighty patients (405 eyes) were included (MOG-ON: n = 57, 20.4%; AQP4-ON: n = 98, 35.0%; seronegative-ON: n = 125, 44.6%). The proportion of eyes with best-corrected visual acuity > 20/25 at the 3-year follow-up was similar between the MOG-ON and seronegative-ON groups; the proportion in both groups was higher than that in the AQP4-ON group (*p* < 0.001). Relapse rates were higher in the MOG-ON and AQP4-ON groups than in the seronegative-ON group (*p* < 0.001). Average retinal nerve fiber layer (RNFL) thickness at 3 years was similar between the MOG-ON and AQP4-ON groups (63.41 ± 13.39 and 59.40 ± 11.46 μm, *p* = 0.476) but both were thinner than the seronegative-ON group (74.06 ± 11.14 μm, *p* < 0.001). Macular ganglion cell-inner plexiform layer (GCIPL) revealed the same pattern. Despite RNFL and GCIPL thinning, the MOG-ON group’s outcome was as favorable as that of the seronegative-ON group, whereas the AQP4-ON group showed unsatisfactory results.

## Introduction

Up until a decade ago, patients with optic neuritis (ON) in China were primarily tested for aquaporin-4 antibodies (AQP4-Abs) due to its high specificity and sensitivity in diagnosing neuromyelitis optica (NMO). Accordingly, patients were divided into two categories: those with multiple sclerosis-related ON (MS-ON), and those with atypical ON, such as that observed in NMO-spectrum disorders (NMOSD)^1–6^. However, some patients would present with recurrent disease despite testing negative for AQP4-Abs, confusing the physicians. With the recent availability of myelin oligodendrocyte glycoprotein antibody (MOG-Ab) testing in China, and the acceptance of MOG-Abs-associated disorders as a unique demyelinating disease of the central nervous system (CNS)^7^, the concept of ON as “atypical” depending on the antibody status, as well as the treatment strategy, has been updated^8^.

For a long time, the epidemiological and clinical characteristics of ON in Asian patients were not as clear as those of MS in Caucasians. The establishment of MOG-Ab as a new diagnostic serological marker for atypical ON has contributed significantly to the understanding of the clinical features and prognosis of MOG-ON in Chinese patients^9–13^.

In this prospective cohort study, we enrolled patients with ON as the primary-onset phenotype in various CNS inflammatory conditions and undertook a 3-year follow-up of these patients. The final visual acuity, relapse rate, and optical coherence tomography (OCT) findings were evaluated to assess the three phenotypes of ON in Chinese patients.

## Results

### Demographic and clinical features

A total of 280 patients (405 eyes) were assessed at the 3-year follow-up visit, including 57 patients (91 eyes) with MOG-ON, 98 patients (148 eyes) with AQP4-ON, and 125 patients (166 eyes) with seronegative-ON. Two patients were seropositive for both MOG-Abs and AQP4-Abs at the first follow-up visit; however, they were eventually seropositive for only AQP4-Abs at the 3-month repeat test and were, thus, assigned to the AQP4-ON group.

The demographic and clinical features of the patients in each group are shown in Table 1. The mean age (mean ± SD) of the patients in the MOG-ON, AQP4-ON, and seronegative-ON groups was 29.26 ± 16.63, 40.64 ± 14.91, and 33.25 ± 13.64 years, respectively. There was no statistically significant difference in age between the MOG-ON and seronegative-ON groups (*p* = 0.423); however, the patients in both these groups were significantly younger than those in the AQP4-ON group (*p* < 0.005). The female-to-male ratio was not significantly different between the MOG-ON and seronegative-ON groups, although a significant female predominance was observed in the AQP4-ON group (*p* < 0.001).

**Table 1:** Demographic and clinical characteristics of patients in the three groups.

	MOG-ON (n = 57, eyes = 91)	AQP4-ON (n = 98, eyes = 148)	Seronegative-ON (n = 125, eyes = 166)	P_1_	P_2_	P_3_
Age, years (mean ± SD)	29.26 ± 16.63	40.64 ± 14.91	33.25 ± 13.64	0.000	0.423	0.002
Female/male (%)	32/25 (1.28)	88/10 (8.8)	78/47 (1.65)	0.000	0.993	0.000
Bilateral	34/57	50/98	41/125	1.000	0.003	0.000
Pain	53/57	81/98	107/125	> 0.05	> 0.05	> 0.05
Disc edema	72/91	105/148	40/166	1.000	0.000	0.000
Course of disease (months)	12.06 ± 25.25	17.16 ± 32.44	3.67 ± 11.51	1.000	0.000	0.000
Relapse rate (%)	44/57 (77.19%)	82/98 (83.67%)	38/125 (30.40%)	1.000	0.000	0.000
Recurrent (n)	1.63 (0–10)	1.27 (0–4)	0.46 (0–3)	1.000	0.000	0.000
**MRI lesions (%)**						
Brain	14/57 (24.56%)	11/98 (11.22%)	27/125 (21.60%)	> 0.05	> 0.05	> 0.05
Spinal cord	0	29/98 (29.59%)	3/125 (2.40%)	0.000	1.000	0.000
**Autoimmune Abs (%)**						
ANA (≥ 1:100)	4/57 (7.02%)	29/98 (29.59%)	15/125 (12.00%)	0.028	1.000	0.000
SSA/SSB	1/57 (1.75%)	18/98 (18.37%)	2/125 (1.60%)	> 0.05	> 0.05	> 0.05
ANCA	0	2/98 (2.04%)	0	> 0.05	0.000	> 0.05

MOG, myelin oligodendrocyte glycoprotein; AQP4, aquaporin-4; ON, optic neuritis; Abs, antibodies; ANA, antinuclear antibody; SSA/SSB, Sjögren syndrome A/B; ANCA, anti-neutrophil cytoplasmic antibody; P_1_, P value MOG-ON compared to AQP4-ON; P_2_, P value MOG-ON compared to seronegative-ON; P_3_, P value AQP4-ON compared to seronegative-ON.

Bilateral optic nerve involvement was higher in the MOG-ON (59.6%) and AQP4-ON (51.0%) groups than in the seronegative-ON group (32.8%) (*p* < 0.005). The patients in the MOG-ON and AQP4-ON groups showed a higher percentage of disc edema than those in the seronegative-ON group (*p* < 0.001).

Among the patients in the MOG-ON, AQP4-ON, and seronegative-ON groups who underwent brain/orbit/spinal MRI, brain lesions that met the McDonald criteria for MS were observed in 24.56%, 11.22%, and 21.60% of patients, respectively (*p* > 0.05).The percentage of patients with spinal cord lesions was high in the AQP4-ON group (29.59%, *p* < 0.001).

The proportion of patients who were positive for concomitant rheumatological antibodies, especially the SSa/SSb and Ro52 for Sjogren's syndrome, was higher in the AQP4-ON group.

### Visual acuity

Visual acuity was measured at presentation and classified into five grades. The number of eyes in each grade classification is shown in Table 2. The best-corrected visual acuity (BCVA) at baseline was significantly different between the APQ4-ON and seronegative-ON group (*p* = 0.043). The final BCVA at the 3-year follow-up showed improvement in all three groups, and the proportion of eyes with BCVA > 0.8 (20/25) in the MOG-ON, AQP4-ON, and seronegative-ON groups was 84.6%, 25.0%, and 75.9%, respectively, thereby being similar in the MOG-ON and seronegative-ON groups (*p* = 0.198), with both being higher than that in the AQP4-ON group (*p* = 0.001).

**Table 2:** Best-corrected visual acuity in the three groups of patients with optic neuritis.

	MOG-ON (*n* = 57, eyes = 91)	AQP4-ON (*n* = 98, eyes = 148)	seronegative-ON (*n* = 125, eyes = 166)	P_1_	P_2_	P_3_
**BCVA (nadir, eyes)**				0.257	0.639	0.043
≥ 20/20	10/91 (10.99%)	4/148 (2.70%)	15/166 (9.04%)			
20/50–20/30	7/91 (7.69%)	20/148 (13.51%)	29/166 (17.45%)			
20/200–20/60	29/91 (31.87%)	23/148 (15.54%)	35/166 (21.08%)			
> CF–< 20/200	25/91 (24.47%)	12/148 (8.11%)	23/166 (13.86%)			
NLP–CF	20/91 (21.98%)	89/148 (60.14%)	64/166 (38.55%)			
**BCVA (3-years, eyes)**				0.001	0.198	0.001
≥ 20/25	77/91 (84.62%)	37/148 (25.00%)	126/166 (75.90%)			
20/50–20/30;	9/91 (9.89%)	17/148 (14.49%)	12/166 (7.23%)			
20/200–20/60	3/91 (3.30%)	25/148 (16.90%)	7/166 (4.22%)			
> CF–< 20/200	2/91 (2.30%)	42/148 (28.38%)	16/166 (9.64%)			
NLP–CF	0	27/148 (18.24%)	5/166 (3.01%)			
Visual improvement	64/91 (70.33%)	48/148 (32.43%)	121/166 (72.89%)	0.000	1.000	0.000

MOG, myelin oligodendrocyte glycoprotein; AQP4, aquaporin-4; ON, optic neuritis; BCVA, best-corrected visual acuity; NLP, no light perception; CF, counting fingers; P_1_, P value MOG-ON compared to AQP4-ON; P_2_, P value MOG-ON compared to seronegative-ON; P_3_, P value AQP4-ON compared to seronegative-ON.

Snellen chart was revised for use in China: 0.8 = 20/25; 0.4 = 20/50; 0.7 = 20/30; 0.3 = 20/60; 0.1 = 20/200; counting fingers; no light perception.

### Relapse rate

The relapse rates in the MOG-ON (77.19%) and AQP4-ON (83.67%) groups were considerably higher than that in the seronegative-ON group (30.40%). The mean recurrent times during the 3-year follow-up period in the MOG-ON and AQP4-ON groups were 1.63 and 1.27, respectively, both of which were higher than that in the seronegative-ON group (0.46, *p* < 0.001).

After 3 years of follow-up, the proportion of patients in the MOG-ON and seronegative-ON groups who developed definitive MS according to the 2017 McDonald criteria^14^ were 12.28% and 13.6%, respectively (*p* = 0.565). The proportion of patients in the AQP4-ON group who developed definitive NMO was 25.5%.

The proportion of patients in the MOG-ON, AQP4-ON, and seronegative-ON groups who stopped their medication were 36/57 (63.16%), 41/98 (41.84%), and 112/125 (89.6%), respectively. The treatment strategy included administration of low-dose prednisone, mycophenolate mofetil, azathioprine, cyclosporine, methotrexate, cyclophosphamide, fingolimod, teriflunomide, interferon-β, rituximab, and immunoglobulin (Table 3). The use of plasma exchange and immunoadsorption therapy was also shown to be effective.

**Table 3:** Treatment agents used in each of the three phenotypes of optic neuritis.

	MOG-ON (*n* = 57)	AQP4-ON (*n* = 98)	seronegative-ON (*n* = 125)
Prednisone	5	12	3
Mycophenolate mofetil	14	6	2
Azathioprine	3	16	0
Cyclosporine	0	10	0
Methotrexate	0	2	1
Cyclophosphamide	0	1	1
Fingolimod	0	0	1
Teriflunomide	0	0	1
Interferon-β	0	0	2
Rituximab	2	13	2
Immunoglobulin	3	4	2
PE or IA	2	7	4
withdrawal medication	36	41	112

MOG, myelin oligodendrocyte glycoprotein; AQP4, aquaporin-4; ON, optic neuritis; PE, plasma exchange; IA, immunoadsorption.

### OCT measurement

**Figure 1:**
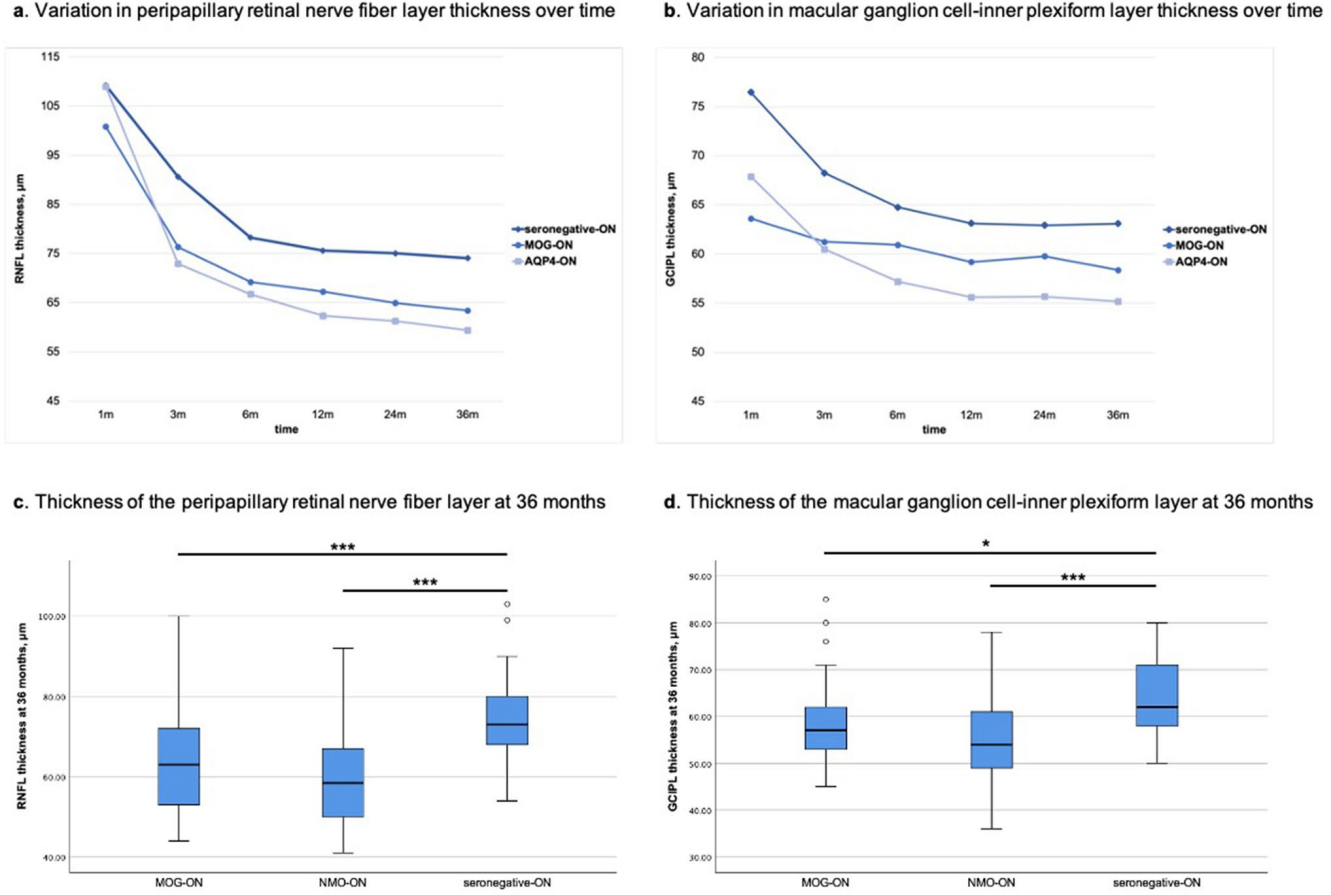
Line graphs and box-and-whisker plots of peripapillary retinal nerve fiber layer (RNFL) and macular ganglion cell-inner plexiform layer (GCIPL) thickness. MOG, myelin oligodendrocyte glycoprotein; ON, optic neuritis; AQP-4, aquaporin 4.

OCT measurements were performed on 60 eyes in the MOG-ON group, 75 eyes in the AQP4-ON group, and 74 eyes in the seronegative-ON group. The follow-up peripapillary retinal nerve fiber layer (RNFL) and macular ganglion cell-inner plexiform layer (GCIPL) thickness measurements are shown in Fig. 1 (Fig. 1a, b, respectively). In the first month following acute onset of ON, there was no significant difference among the three groups regarding RNFL and GCIPL thickness. However, both RNFL and GCIPL thickness rapidly decreased until 6 months following the acute onset, and then remained almost stable, regardless of the phenotype. The average RNFL thicknesses in the MOG-ON, AQP4-ON, and seronegative-ON groups were 63.41 ± 13.39, 59.40 ± 11.46, and 74.06 ± 11.14 μm, respectively, with no significant difference between the MOG-ON and AQP4-ON groups; the RNFL was significantly thinner in these two groups than in the seronegative-ON group (*p* < 0.001) (Fig. 1c). RNFL thinning occurs predominantly in the temporal peripapillary quadrant than in the other quadrants.

The macular GCIPL thickness showed a similar pattern, wherein the GCIPL thickness decreased significantly in the MOG-ON and AQP4-ON groups compared with that in the seronegative-ON group (*p* < 0.001). There were no significant differences in the GCIPL thickness between the MOG-ON and AQP4-ON groups (Fig. 1d). However, GCIPL thinning did not show any quadrant predilection in this study.

## Discussion

Testing of MOG-Abs and AQP4-Abs in patients with new-onset ON has changed the classification and clinical process of ON, as well as the understanding of ON phenotypes in China^1,15^. Depending on the serological biomarkers, it is possible to evaluate and follow-up the prognosis of different types of ON.

**Figure 2:**
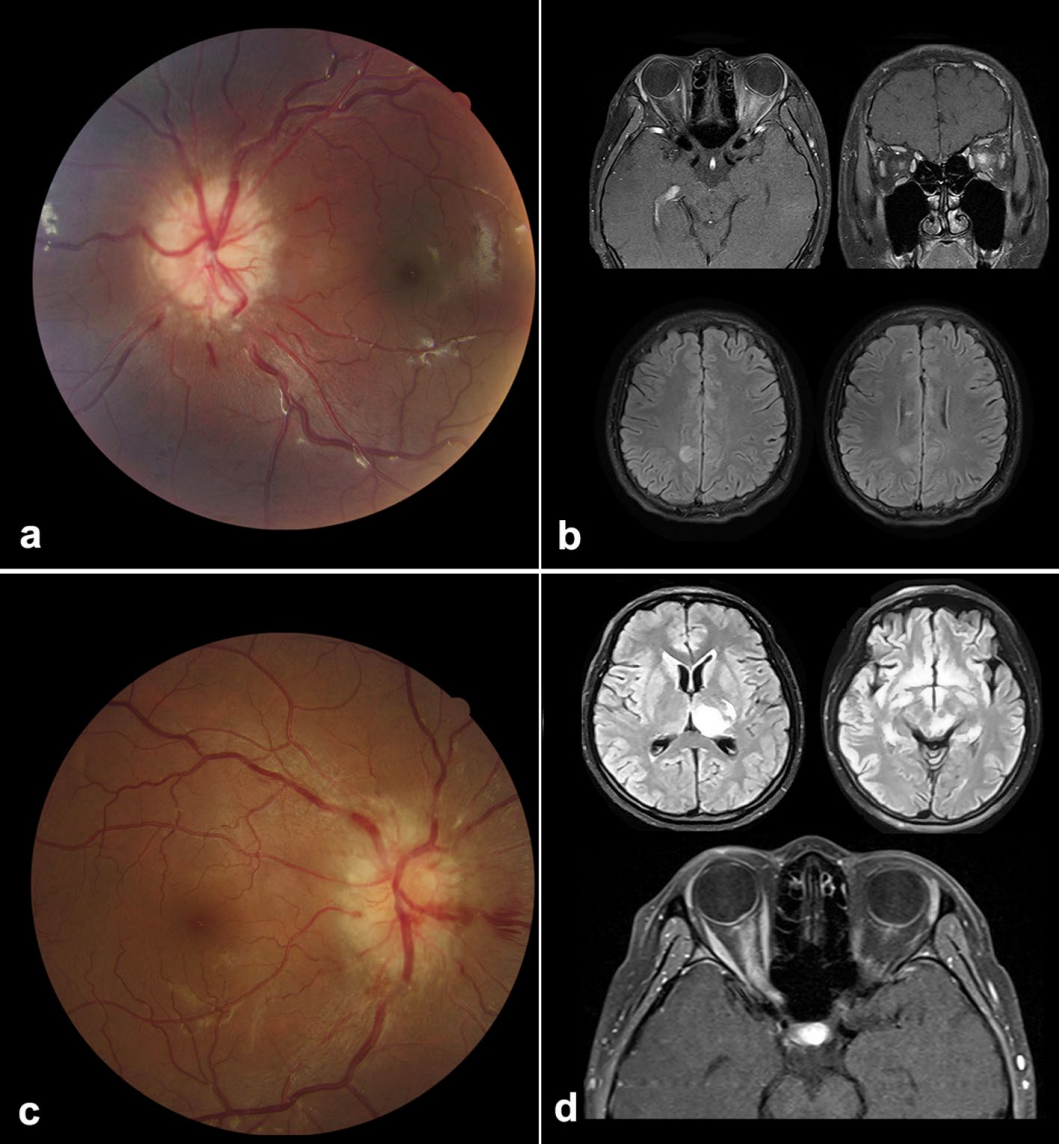
Patients who had a positive result for myelin oligodendrocyte glycoprotein antibodies presenting with brain lesion without diagnosis. (**a**, **b**) Images of a 12-year-old boy presenting with acute visual loss in the left eye for 10 days. One month ago, he experienced sudden loss of consciousness and a seizure. Fundoscopy (**a**) shows obvious edema of the optic disc, and brain and orbit magnetic resonance imaging (MRI) (**b**) shows enlarged and enhanced left optic nerve with brain lesions, indicating acute disseminated encephalomyelitis-like demyelinating disease, which was the probable cause for the seizure. (**c**, **d**) Images of a 30-year-old woman presenting with acute visual decrease in the right eye for 2 weeks. One month ago, she complained of weakness in her right body, while brain MRI showed a midbrain lesion, which recovered spontaneously. Fundoscopy (**c**) shows a severe swelling of the optic disc, and orbit MRI (**d**) shows extensive enhancement of the nerve.

Our prospective 3-year study included a considerably large cohort of Chinese patients with ON. Surprisingly, > 20% of patients in our cohort tested positive for serum MOG-Abs and were characterized by a distinct set of clinical features, such as young-age onset, bilateral involvement, severe disc swelling, longitudinally extensive nerve and sheath enhancement in MRI imaging, and good recovery, albeit with a high relapse rate^7,9,16^. Since our cohort also included pediatric patients aged < 18 years old, the mean age of the MOG-ON group was much lower than that of the other studies and that reported for adult patients with MOG demyelinating disorders^7,11,12^. Some pediatric patients presented with fever, headache, and seizures before vision loss, without a definitive diagnosis; however, during the workup for acute ON, the MRI scan showed some subclinical demyelinating lesions in the brain and optic nerves, which was consistent with acute disseminated encephalomyelitis (ADEM) (Fig. 2a). Furthermore, ON could be viewed as a window to the brain^17^. It is easy for a neuro-ophthalmologist to diagnose MOG-associated disorders when the optic nerve is involved, regardless of the presence of previous brain lesions, which may be confusing for neurologists (Fig. 2b).

**Figure 3:**
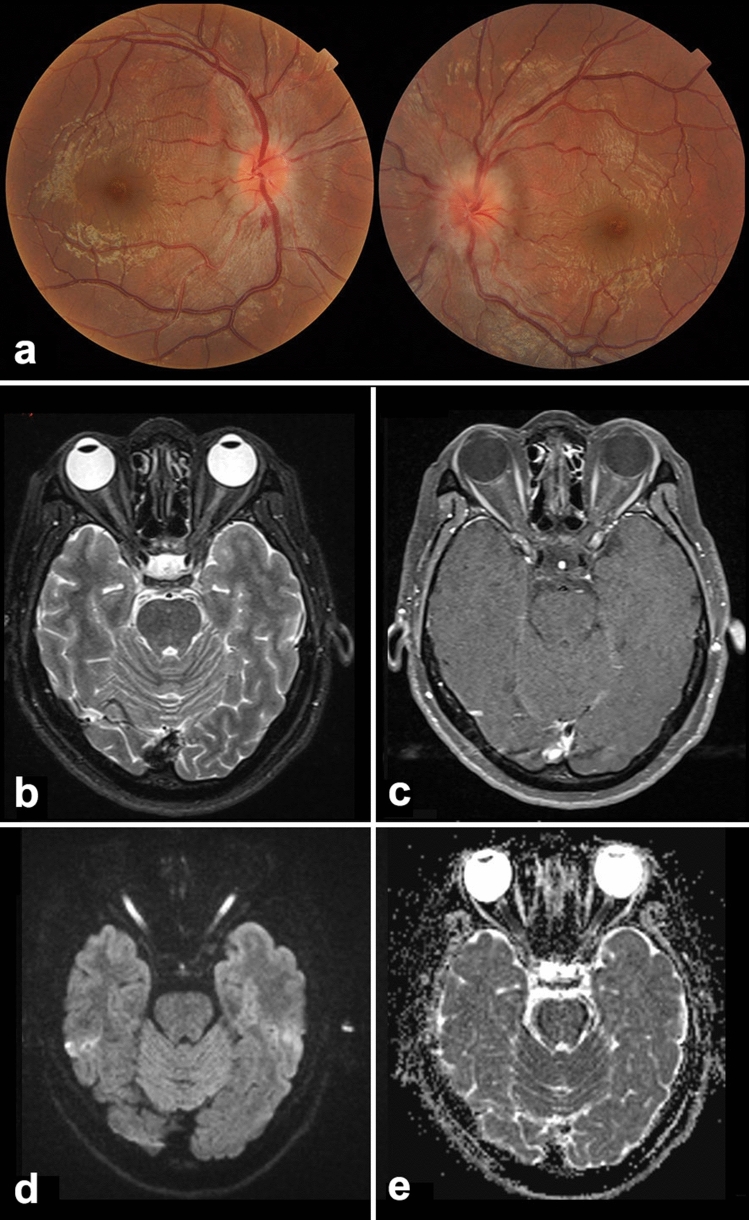
A 20-year-old young man with acute profound bilateral visual loss, seronegative for myelin oligodendrocyte glycoprotein and aquaporin 4 antibodies. (**a**) The disc is swollen bilaterally with hemorrhage; (**b**–**e**) Orbit magnetic resonance imaging (MRI) at the acute stage showing mild hyperintensity of the optic nerves on T2-weighted imaging (WI) (**b**), no enhancement on T1WI after contrast (**c**); diffusion-weighted MRI (**d**) showing extreme hyperintensity of bilateral optic nerves with reduced apparent diffusion coefficient (**e**) value, indicating severe damage of the nerve. The best-corrected visual acuity at the 3-year follow up was 20/400 OU.

Our study showed that the final BCVA of the patients with ON at the 3-year follow-up was favorable in the MOG-ON and seronegative-ON groups, despite the several recurrences observed in the MOG-ON group. Nearly 85% of eyes in the MOG-ON group and > 75% eyes in the seronegative-ON group recovered to > 20/25 visual acuity. In contrast, the BCVA was < 20/200 in more than one-third of the patients in the AQP4-ON group. There were also some exceptional cases in our cohort wherein patients with bilateral involvement and severe ON tested negative for both MOG-Abs and AQP4-Abs and had a very poor prognosis for visual recovery. Acute-stage orbit MRI in these patients showed hyperintensity on the diffusion-weighted image instead of optic nerve enhancement after intravenous gadolinium administration (Fig. 3). The exact etiology for MOG-ON remains unknown; however, we presume that some unknown infectious toxic processes or unknown antibodies attacking the optic nerve axons might be involved, rather than the current demyelinating mechanisms involved in seronegative-ON cases^18,19^.

In our previous study, the recurrence frequency in patients with MOG-ON was 2.71 during a 3-month follow-up^9^; this was reduced to 1.63 during the 3-year follow-up in this study. Administration of a slowly tapering dose of steroids such as prednisone and adding immunosuppressive agents such as mycophenolate mofetil (MMF) effectively reduced the relapse rate; even if recurrence occurred, the second episode of onset seemed milder than the first one. After 3 years of follow-up in this study, the medications were stopped in nearly half of the patients in the MOG-ON group; however, more than two-thirds of those in the AQP4-ON group still require long-term administration of low-dose prednisone or immunosuppressive agents. In contrast, the medications were stopped in most patients in the seronegative-ON group. The most widely used medication for recurrent prophylaxis in MOG-ON is MMF, which was shown to have good efficacy and tolerance in Chinese patients^20^. In contrast, Azathioprine, which is routinely used as the first-line immunosuppressive treatment for NMO in Western countries, is less commonly utilized in China due to its association with hepatic lesions. Rituximab, which is not covered by medical insurance in China, cannot be widely used for frequent recurrent MOG-ON and NMO-ON. A low dose of rituximab with shortened intervals has been reported to yield good results in some centers^21,22^. We also observed more frequent relapses in some patients with NMO after repeated use of low-dose rituximab at 1-month intervals. Further long-term studies will be needed before this regimen becomes widely accepted. Immunoglobulin, as another biological agent, is mainly used in children with high titers of MOG-Abs or ADEM demyelination of the brain and has also been recommend by the international guidelines for pediatric patients^23^.

Immunoadsorption (IA) therapy is a widely recommended escalation treatment for steroid-resistant ON. In our cohort, 13 patients received add-on IA therapy (MOG-ON: n = 2; AQP4-ON: n = 7; seronegative-ON: n = 4). Following IA therapy, the BCVA improved in 9 of 13 patients (69.2%), and the antibody titer decreased in 7 of 13 patients (53.8%). Our preliminary results showed that IA therapy can improve the visual function in patients with severe ON who are resistant to steroid treatment. A shorter disease course was associated with a higher possibility of improvement in visual acuity, and no severe side effects were observed in our cohort.

The proportion of patients in the MOG-ON and seronegative-ON groups who developed definitive MS according to the 2017 McDonald criteria^14^ after 3 years of follow-up was 12.28% and 13.6%, respectively. One-quarter of patients in the AQP4-ON group developed definitive NMO after 3 years of follow-up. The high transfer rate made it very important for ophthalmologists to treat patients who experienced a first attack of ON but had positive AQP4-Abs.

OCT measures such as optic disc RNFL thickness and macular GCIPL thickness are routinely utilized for evaluating optic nerve atrophy following the attack and, to a certain extent, help predict visual function^24–26^. Our data showed that loss of peripapillary RNFL and macular GCIPL in the MOG-ON group was as profound as that in the AQP4-ON group, even though the BCVA recovery was excellent in the former group. Although there was no statistically significant difference among the three groups in the first month after the acute-onset ON, the thickness of both RNFL and GCIPL decreased rapidly during the next 6 months and then stabilized until the 3-year follow-up. The thinning of the RNFL and GCIPL in the MOG-ON and AQP4-ON groups was significantly greater than that in the seronegative-ON group at the 3-year follow-up. Nevertheless, there were no statistically significant differences in RNFL and GCIPL thickness between the MOG-ON and AQP4-ON groups. A marked reduction in the RNFL and GCIPL thicknesses can be observed in patients with NMOSD with proportional disc atrophy and poor visual acuity^27,28^. Although patients in the MOG-ON group showed excellent recovery in terms of visual acuity, the mechanism underlying this phenomenon is not clear; demyelination of the nerve rather than damage of the axon might partially explain the contradiction.

Although our cohort included a relatively large sample, our study was performed in a single center, which might limit its generalizability. Furthermore, comorbidities like diabetes and hypertension which could have influenced the results should be added to limitation. Thus, multicenter studies and standardized recruitment criteria will be needed for further research.

This 3-year follow-up study on Chinese patients with different ON phenotypes revealed that atypical ON, including MOG-ON and AQP4-ON, accounts for more than half of all ON cases. Despite the obvious thinning of the RNFL and GCIPL, the outcome of MOG-ON was as favorable as that of seronegative-ON, whereas that for AQP4-ON was unsatisfactory. The relapse rate of MOG-ON was as high as that of AQP4-ON, and more than one-quarter of patients in the latter group developed definitive NMO after 3 years. Therefore, testing for serum biomarkers such as MOG-Abs and AQP4-Abs is important when patients experience the first attack of ON, since the evaluation and treatment paradigms depend significantly on the serum status and phenotypes.

## Methods

### Patient selection

This study was an extended follow-up of our previous study^9^. Between January 2015 and March 2017, patients with newly diagnosed acute-onset ON were sequentially enrolled for this study in the Ophthalmology Department of the Eye Ear Nose and Throat Hospital in Shanghai, China. This study complied with the tenets of the Declaration of Helsinki. All experiments were performed in accordance with the relevant guidelines and regulations and were approved by the ethics committee of the Eye Ear Nose and Throat Hospital (KJ2011-04). All study participants provided written informed consent, and the consent form was signed by a guardian for participants aged < 18 years. The diagnosis of all patients was confirmed by both neuro-ophthalmologists and neurologists. The inclusion criteria for diagnosing ON were based on the Optic Neuritis Treatment Trial, with some revision, as follows^29^: (1) acute unilateral or bilateral new-onset visual decrease and/or visual field defect within 1 month; (2) a relative afferent pupillary defect (if the damage was unequal) and/or abnormal visual field tests; (3) ancillary laboratory test results showing no evidence of infectious, hereditary, ischemic, toxic, and metabolic etiology; (4) ability to perform brain/orbit MRI to assess the lesion in the optic nerve and brain and rule out compression.

The exclusion criteria included: (1) absence of serum MOG-Abs and AQP4-Abs; (2) refusal to sign the consent form; (3) incomplete clinical data or inability to remain in the study for a 3-year follow-up.

**Figure 4:**
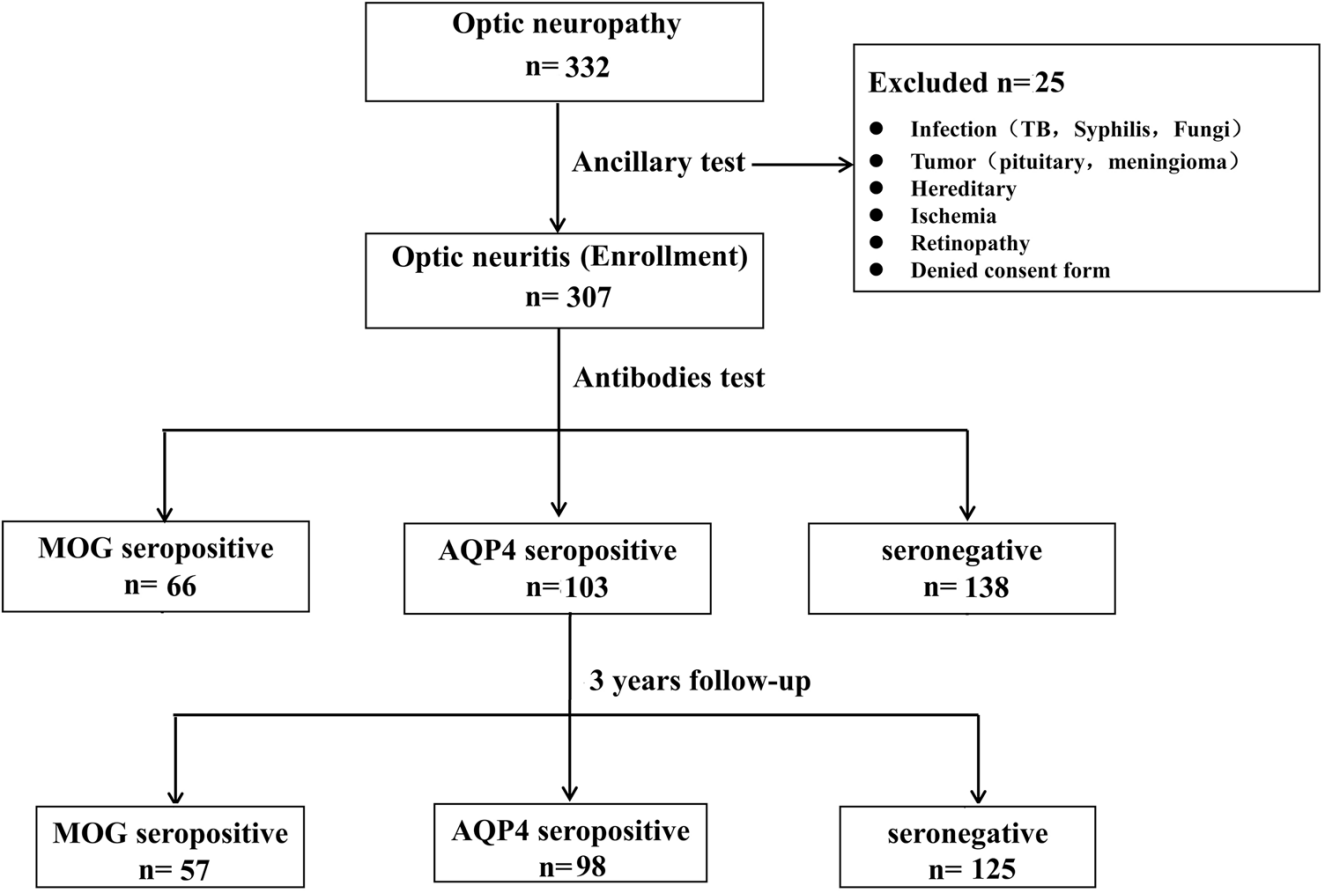
Study diagram and grouping of patients. TB, tuberculosis; MOG, myelin oligodendrocyte glycoprotein; ON, optic neuritis; AQP-4, aquaporin 4.

As shown in Fig. 4, a total of 332 patients with presumed ON were referred to neuro-ophthalmologists for evaluation. After routine ophthalmological examinations, ancillary laboratory tests, and brain/orbit imaging, 25 patients were excluded due to infection, compressive optic neuropathy, hereditary optic neuropathy, anterior ischemic optic neuropathy, and retinopathy; finally, 307 patients were enrolled in the study. The cohort was divided into three groups according to the serum test performed on the patient: namely, the MOG-ON, AQP4-ON, and seronegative-ON groups. The clinical course of all patients was followed-up for at least 3 years and, finally, 280 patients with complete clinical data were evaluated (Fig. 4).

### Demographic data and ophthalmological examinations

Data regarding age, sex, disease course, laterality of the eye, and ophthalmological examination of the fundus were collected.

As the main outcome, we measured the BCVA using the revised standard Snellen chart in China and grouped it into five grades as follows: 0.8 or better (≥ 20/25); 0.4–0.7 (20/50–20/30); 0.1–0.3 (20/200–20/60); < 0.1 (> counting fingers); counting fingers (no light perception).

### Serum antibodies tests

All enrolled patients underwent serum antibody tests, including a routine blood test. Serum samples were tested for MOG-Abs and AQP4-Abs using a cell-based assay protocol. The samples were tested using a fixed cell-based indirect immunofluorescence test in one of the branches of Euroimmun Medical Diagnostic Laboratory in China (EUROIMMUN AG, Lübeck, Germany). Full-length human MOG and AQP4 isoform M1-transfected HEK293 cells were used in this test.

### Optical coherence tomography

Spectral-domain OCT was performed using 3D disc, optic nerve head, and GCIPL protocols provided by RTVue-100, version 4.0.7.5 (Optovue Inc., Fremont, CA). The peripapillary RNFL thickness was measured automatically using the RNFL 3.45 scanning mode, for which four circular scans (1024 A-scans/scan) are acquired 3.45 mm from the center of the optic disc. The GCIPL scan technique provides inner-retinal thickness values, which include the ganglion cell layer and inner plexiform layer. The GCIPL within the central 6-mm-diameter area of the macular was calculated. The thickness of the optic disc RNFL and macular GCIPL were measured at the 1-, 3-, 6-, 12-, 24-, and 36-month follow-up visits.

### Statistical analysis

The demographic variants were described and compared among the three groups. The Kruskal−Wallis test was used for non-parametric comparisons, and the Student’s *t*-test was used for parametric comparisons. The rank-sum test for non-parametric data was used for evaluating visual acuity. Considering the intra-subject correlations, generalized estimating equation models were used for the OCT data. A *P* value < 0.05 was considered statistically significant. All analyses were carried out using IBM SPSS statistics for Windows, version 23.0.0.0 (IBM Corp., Armonk, New York, USA).

### Consent for publication

Written informed consent for publication was obtained from all participants.

## Data Availability

The datasets or analyzed during the current study are available from the corresponding author on reasonable request.
